# Healthcare Workers’ Assessment of a Visual Triage System (VTS)

**DOI:** 10.7759/cureus.49910

**Published:** 2023-12-04

**Authors:** Fahad Alsalhi, Imen Sohaibani, Abdulelah Alshammari, Ahmed Al-Amri, Own Al-Kathiri, Mazen Altamimi, Malak Alharbi, Mohammed Altamimi, Mohannad Khayat, MH Rajab

**Affiliations:** 1 Public Health, Alfaisal University College of Medicine, Riyadh, SAU; 2 Public Health, Ministry of Health, Riyadh, SAU; 3 Public Health Operation Center, Ministry of Health, Riyadh, SAU; 4 Radiology, Ministry of Health, Rafha, SAU; 5 Infectious Disease, Ministry of Health, Riyadh, SAU; 6 Pharmacology and Therapeutics, King Fahd Security College, Riyadh, SAU; 7 Epidemiology and Public Health, Alfaisal University, Riyadh, SAU

**Keywords:** middle east respiratory syndrome (mers-cov), healthcare workers (hcws), emergency department (ed), visual triage system (vts), covid-19

## Abstract

Overcrowding and extended waiting times in the emergency department (ED) can pose a significant risk of COVID-19 transmission from patients to healthy individuals. In 2017, the Saudi Ministry of Health (MOH) introduced a visual triage system (VTS) with scoring to notify healthcare workers (HCWs) in EDs about the Middle East respiratory syndrome coronavirus (MERS-CoV) infection risk. During the COVID-19 pandemic, the MOH employed a VTS to classify patients according to their potential risk of COVID-19 infection upon their admission to the ED. Suspected patients were then directed along specific pathways to reduce their contact with healthy individuals. This study assessed HCWs’ satisfaction with the VTS in the ED of two major government hospitals within the Riyadh region. Additionally, it assessed HCWs’ perceptions of VTS effectiveness. This study used a cross-sectional, observational design and relied on surveys for data collection. A total of 127 participants completed the survey, of which 87 (68.5%) were based in the EDs of the two hospitals. Among the ED participants, 18.1% expressed satisfaction with the VTS, 46.4% were neutral, and 33.1% reported dissatisfaction. ED participants provided feedback on the system’s effectiveness, with 24.1% finding it effective, 66.7% considering it somewhat effective, and 9.2% deeming it ineffective. Of the total (127) study participants (70.1%) reported that the HCWs required better training to effectively implement the VTS infection control plans for suspected cases. Fewer than half of the participants (35.4%) deemed the time spent by VTS personnel to identify COVID-19 cases to be reasonable, whereas 22% found it too short and 27.6% considered it too long. Of the total 127 participants, 63% reported that language differences between patients and HCWs constituted barriers to the effective application of the VTS. Our study findings indicated that most ED participants had a neutral outlook on their satisfaction with the VTS and a neutral perspective on the effectiveness of VTS, viewing it as only somewhat effective. Reported weaknesses and key obstacles to the successful implementation of the VTS included language barriers. and insufficient training for HCWs, and unclear VTS pathways. The reported strengths of the VTS included its effectiveness in reducing crowds and identification of COVID-19 patients.

## Introduction

The concept of triage, stemming from the French verb “trier,” plays a pivotal role in medical practice [1-3]. Triage originated in scenarios marked by constrained resources and time limitations, in which efficient decision-making becomes paramount [4]. Triage is a systematic framework for categorizing casualties to optimize life-saving outcomes. The significance of this concept transcends battlefields and extends to emergency departments (EDs), where efficiency is equally crucial [2,5]. The Manchester Triage System (MTS), developed in Manchester, UK, more than two decades ago, is among the most widely adopted triage systems [6]. It employs a 5-point scale to prioritize patients in the ED, streamlining timely and accurate decision-making in alignment with the core purpose of triage in medical settings [7].

Another notable system is the Canadian Triage and Acuity Scale (CTAS), introduced in 1999 for hospital EDs in Canada [8]. Using a 5-point scale, CTAS prioritizes patients based on the severity of their condition, providing a standardized approach to triage. Its validation in various countries, including Saudi Arabia, underscores its reliability among ED triage nurses, facilitating effective patient management [9].

Preparation for emerging diseases is a fundamental requirement for healthcare facilities seeking to enhance medical safety. The COVID-19 pandemic serves as a stark reminder of this necessity, as healthcare infrastructures globally face unprecedented strains. Notably, EDs have borne the brunt of these challenges. Considering the high communicability of COVID-19, EDs emerged as potential hubs for disease transmission, demanding heightened preparedness and vigilance of a healthcare worker (HCW) at entry points [10]. The risk of contagion between patients, particularly in overcrowded hospitals, prompted health policymakers in Saudi Arabia to introduce guidelines mandating visual triage stations at ED entrances [11]. This practice has since become compulsory for all Saudi hospitals.

In 2017, the Saudi Ministry of Health (MOH) introduced a visual triage system (VTS) with scoring to notify HCWs in EDs about the risk of Middle East respiratory syndrome coronavirus (MERS-CoV) infection [11]. During the pandemic, the MOH employed a VTS to classify patients according to their potential risk of COVID-19 infection upon their admission to the ED. Suspected COVID-19-positive patients were then directed along specific pathways to reduce their contact with healthy individuals [11].

Moreover, the infection prevention and control (IP&C) departments at these hospitals monitored and evaluated the level of COVID-19 infection risk based on VTS effectiveness in EDs.

Diverse triage methods for patients infected with COVID-19 reflect variations in disease symptoms across countries. The optimal triage system is intended to sort and classify patients during outbreaks of infectious disease. The visual triage station at the ED entrance involves assessing patients using a numerical scale checklist [12]. Trained nurses conduct interviews and assessments; patients scoring ≥4 are directed to a respiratory waiting area after hand hygiene procedures and wearing surgical masks. Doctors subsequently assess these patients in designated respiratory zones. These stations have proven immensely useful in the timely detection of suspected cases, averting cross-transmission, and preventing hospital outbreaks [12].

Our study investigated the effectiveness and satisfaction levels associated with the VTS from the viewpoint of HCWs during the COVID-19 pandemic in two prominent hospitals in Riyadh. By directly soliciting feedback from frontline HCWs, we have gained valuable insights into performance and the general satisfaction levels of VTS among HCWs.

## Materials and methods

Using a survey-based cross-sectional, observational approach, the study focused on HCWs employed in the EDs of two prominent hospitals in Riyadh. We also collected data on infection control HCWs since part of their routine operations is to continuously monitor and assess the effectiveness of the VTS introduced in the EDs. There are two main points based on which the assessment was done in this study, effectiveness of the VTS and satisfaction with the VTS. For each point, we included several questions in the survey. The VTS is routinely assessed as per the MOH announced schedule. The triage is done usually by the emergency room (ER) nurses. The inclusion criteria encompassed HCWs with a minimum of three months of experience in dealing with the VTS.

We used an online self-administered questionnaire available in two versions, English and Arabic. Before data collection, the study questionnaire was validated by physicians from the MOH’s IP&C Department to ensure the clarity and appropriateness of the included questions.

The questionnaire consisted of three sections. The first section collected information on participant demographics, including age, gender, department, hospital, position, and nationality. The second section consisted of questions aimed at evaluating participants’ satisfaction with the VTS and its overall effectiveness. Responses to the VTS satisfaction question were classified into three categories: satisfied, neutral, and dissatisfied. In the third section, open-ended questions were incorporated to gather participants’ responses regarding both the advantages and disadvantages they perceived in using the VTS.

The minimum required sample size for this study was determined to be 113 participants out of a total population of 350 HCWs across the two studied hospitals, with a response distribution estimated at 50%, a desired statistical power of 80%, and a permissible margin of error of 5%.

Statistical analysis was conducted using Statistical Package for the Social Sciences (SPSS) version 26 (IBM Corp., Armonk, NY), designed for Microsoft Windows. Categorical data were presented as frequencies (number of cases) and corresponding percentages. To assess the differences between subgroups, statistical comparisons were made using the Chi-squared or Fisher’s exact test, wherever appropriate. P-values of less than 0.05 were considered statistically significant.

Before commencing the study, we obtained an institutional review board (IRB) exemption to perform this research from the IRB within Health Cluster One of Riyadh City. All data collected from study participants were treated with utmost confidentiality, ensuring their anonymity.

## Results

This study assessed HCWs’ satisfaction with the VTS in the ED department of two major hospitals in the Riyadh region. Additionally, it assessed their perceptions of VTS effectiveness.

Table 1 provides an overview of the demographics of study participants. Out of the 127 HCWs included, 74% (94) fell within the age group ranging from 25 to 39 years. Among the participants, approximately half (53.5% (68)) were ER nurses, 31.5% (40) were IP&C specialists, and 15% (19) were ER physicians. Furthermore, 68.5% (87) of the participants were employed in the EDs of the two studied hospitals.

**Table 1:** Demographic characteristics of study participants (n = 127) ER, emergency room; IP&C, infection prevention & control

Parameters	Category	Frequency	Percentage (%)
Age	25–39 years	94	74
	40–60 years	33	26
Position	ER physician	19	15
	ER nurse	68	53.5
	IP&C staff	40	31.5
Department	Emergency	87	68.5
	IP&C	40	31.5
Gender	Male	51	40.2
	Female	76	59.8
Nationality	Saudi	67	52.8
	Non-Saudi	60	47.2
Language of the questionnaire	English	48	38
	Arabic	79	62

Regarding gender and nationality, most of the participants were female (59.8% (76)) and of Saudi nationality (52.8% (67)). Additionally, the questionnaire was primarily administered in Arabic, with 62% (79) of participants responding in this language.

Table 2 illustrates the overall satisfaction of all study participants (N=127) with the VTS. Specifically, 18.1% (23) of participants expressed satisfaction, 46.4% (59) had a neutral opinion, and 33.1% (42) were dissatisfied. Regarding effectiveness, most participants (56.7%) considered the system somewhat effective in the early detection and control of COVID-19 cases in the ED, 26% (33) deemed it effective, and 14.2% (18) found it to be ineffective. Furthermore, a significant proportion of participants (70.1% (89)) believed that the VTS staff were inadequately trained, and 63% (80) recognized that language differences between HCWs and patients posed a barrier to VTS effectiveness. Moreover, more than one-third of the respondents (35.4% (45)) reported that the time spent by the VTS in identifying COVID-19 cases was reasonable.

**Table 2:** Healthcare workers’ (n = 127) evaluation of the VTS VTS, visual triage system

Parameters	Category	Frequency	Percentage (%)
Satisfaction with VTS	Satisfied	23	18.1
	Neutral	59	46.4
	Dissatisfied	42	33.1
	No opinion	3	2.4
Effectiveness of VTS	Effective	33	26
	Somewhat effective	72	56.7
	Ineffective	18	14.2
	No opinion	4	3.1
Healthcare workers are adequately trained to detect suspected cases	Agree	33	26
	Disagree	89	70.1
	No opinion	5	3.9
Time to identify suspected cases	Too short	28	22
	Reasonable	45	35.4
	Too long	35	27.6
	No opinion	19	15
Language barriers	Agree	80	63
	Disagree	37	29.1
	No opinion	10	7.9

Table 3 presents a comprehensive view of the strengths and weaknesses of the VTS as perceived by HCWs in the study. The most commonly reported weaknesses of the VTS were the presence of unclear pathways, the absence of well-trained VTS physicians, and an unclear checklist. Reported strengths of the VTS included its effectiveness in organizing and reducing crowd congestion and its ability to triage emergency cases, facilitating quicker follow-up on such cases.

**Table 3:** Reported VTS strengths and weaknesses and suggestions for improvement (n = 127) Some participants provided more than one answer VTS, visual triage system

VTS parameter	Category	Frequency	Percentage (%)
Weaknesses	Unclear pathways	59	46.5
	Lack of trained physicians	29	22.8
	Unclear checklist	25	19.7
	Time-consuming	17	13.4
	Complicated	11	8.7
	Useless	11	8.7
	Increased crowdedness	6	4.7
Strengths	Organize and reduce crowds	20	20.4
	Identification of COVID-19 patients	21	21.4
	Optimizing patient pathway	14	14.2
	Efficiency in responding to emergency	13	13.2
	Other	17	17.3
Suggestion for improvement	Increase employee training	46	46.9
	Triage by doctors and nurses only	13	13.3
	Define specific triage points in the reception of emergency departments and clinics	8	8.2
	Define specific pathways	6	6.1
	Preliminary triage by nurses and final triage by doctors	4	4.1
	Other	2	2

The most frequently suggested improvements included the need for adequate and ongoing training for the VTS staff, involving both triage processes and checklists. Additionally, it was recommended that VTS be exclusively conducted by specialized doctors and nurses. Other suggestions included starting initial screening by nurses and final screening by doctors, establishing defined triage points in the reception areas of EDs and clinics, and specifying visual triage pathways.

The data regarding language barriers are illustrated in Table 4. More than two-thirds (70.1% (61)) of HCWs in the ED found that the language differences between VTS HCWs and patients presented a significant barrier to the effective application of the VTS. This sentiment was shared by only 47.5% (19) of their colleagues in the infection control department (p = 0.014).

**Table 4:** Analysis of the language differences between VTS healthcare workers and patients VTS, visual triage system; ER, emergency room; IP&C, infection prevention & control

Factors		Language differences between VTS healthcare workers and patients presented barriers to the effective application of the VTS		p-value
	Categories	Agree	Disagree	
Gender	Female	46 (60.5%)	30 (39.5%)	0.482
	Male	34 (66.7%)	17 (33.3%)	
Age	25–39	63 (67%)	31 (33%)	0.112
	40–60	17 (51.5%)	16 (48.5%)	
Nationality	Non-Saudi	39 (65%)	21 (35%)	0.657
	Saudi	41 (61.2%)	26 (38.8%)	
Department	Infection Control	19 (47.5%)	21 (52.5%)	0.014
	Emergency	61 (70.1%)	26 (29.9%)	
Position	ER nurse	49 (72.1%)	19 (27.9%)	0.017
	ER physician	13 (68.4%)	6 (31.6%)	
	IP&C staff	18 (45%)	22 (55%)	

Moreover, 72.1% (49) of ER nurses found that language differences were significant barriers to the VTS (p = 0.017). This sentiment was shared by 68.4% (13) of ER physicians and 45% (18) of IP&C staff.

Table 5 shows data about respondents’ perceptions of the effectiveness of VTS in detecting and controlling COVID-19 cases. For instance, a higher proportion of IP&C staff (37.5% (15)) considered VTS to be effective (p < 0.001) than ER physicians (21.1% (4)) and ER nurses (20.6% (14)). Moreover, the workers in the infection control department were more likely (30% (12)) to consider the VTS to be effective than those who worked in the ED (24.1% (21)) (p < 0.001). Saudi participants (34.3% (23)) were more likely to consider the VTS to be effective than non-Saudi participants (16.7% (10)), with a p-value of 0.021.

**Table 5:** Perceived VTS effectiveness in the detection and control of COVID-19 cases VTS, visual triage system; ER, emergency room; IP&C, infection prevention & control

		Effective	Somewhat effective	Ineffective	p-value
Gender	Female	18 (23.7%)	42 (55.3%)	16 (21.1%)	0.373
	Male	15 (29.4%)	30 (58.8%)	6 (11.8%)	
Age (years)	25–39	24 (25.5%)	59 (62.8%)	11 (11.7%)	0.011
	40–60	9 (27.3%)	13 (39.4%)	11 (33.3%)	
Nationality	Non-Saudi	10 (16.7%)	35 (58.3%)	15 (25%)	0.021
	Saudi	23 (34.3%)	37 (55.2%)	7 (10.4%)	
Department	Infection control	12 (30%)	14 (35%)	14 (35%)	<0.001
	Emergency	21 (24.1%)	58 (66.7%)	8 (9.2%)	
Position	ER nurse	14 (20.6%)	49 (72.1%)	5 (7.4%)	<0.001
	ER physician	4 (21.1%)	12 (63.2%)	3 (15.8%)	
	IP&C staff	15 (37.5%)	11 (27.5%)	14 (35%)	

As indicated in Table 6, 38.8% (26) of Saudis considered hospital HCWs to be well-trained compared with 11.7% (7) non-Saudis (p < 0.001). However, factors such as age, gender, departments, and positions had no significant impact on perceptions of whether HCWs were well trained. As indicated in Table 6, 38.8% (26) of Saudis considered hospital HCWs to be well-trained compared with 11.7% (7) non-Saudis (p < 0.001). However, factors such as age, gender, departments, and positions had no significant impact on perceptions of whether HCWs were well trained.

**Table 6:** Healthcare workers in the hospital are well-trained ER, emergency room; IP&C, infection prevention & control

Factors		Healthcare workers in the hospital are well-trained		p-value
	Categories	Agree	Disagree	
Gender	Female	19 (25%)	57 (75%)	0.758
	Male	14 (27.5%)	37 (72.5%)	
Age	25–39	28 (29.8%)	66 (70.2%)	0.099
	40–60	5 (15.2%)	28 (84.8%)	
Nationality	Non-Saudi	7 (11.7%)	53 (88.3%)	<0.001
	Saudi	26 (38.8%)	41 (61.2%)	
Department	Infection control	7 (17.5%)	33 (82.5%)	0.139
	Emergency	26 (29.9%)	61 (70.1%)	
Position	ER nurse	18 (26.5%)	50 (73.5%)	0.985
	ER physician	5 (26.3%)	14 (73.7%)	
	IP&C staff	10 (25%)	30 (75%)	

As shown in Table 7, 52.2% (35) of Saudis found that the VTS identified a suspected COVID-19 case in a reasonable time compared with 16.7% (10) of non-Saudis (p < 0.001). Meanwhile, factors such as age, gender, position, and working departments had no significant influence on perceptions of the ability of VTS to detect new cases of COVID-19 in a reasonable time.

**Table 7:** Time spent to identify a suspected COVID-19 case VTS, visual triage system; ER, emergency room; IP&C, infection prevention & control

Factors		Time spent for VTS to identify a suspected COVID-19 case		p-value
	Categories	Too long or too short	Reasonable	
Gender	Female	52 (68.4%)	24 (31.6%)	0.268
	Male	30 (58.8%)	21 (41.2%)	
Age	25–39	60 (63.8%)	34 (36.2%)	0.769
	40–60	22 (66.7%)	11 (33.3%)	
Nationality	Non-Saudi	50 (83.3%)	10 (16.7%)	<0.001
	Saudi	32 (47.8%)	35 (52.2%)	
Department/Workplace	Infection control	29 (72.5%)	11 (27.5%)	0.205
	Emergency	53 (60.9%)	34 (39.1%)	
Position	ER nurse	42 (61.8%)	26 (38.2%)	0.682
	ER physician	12 (63.2%)	7 (36.8%)	
	IP&C staff	28 (70%)	12 (30%)	

## Discussion

The current study sought to assess the level of satisfaction with and effectiveness of the MOH’s VTS among HCWs in the emergency departments of two major hospitals within the Riyadh region. This study showed that nearly half of the respondents (48.8%) were neutral, whereas 33.1% were dissatisfied, and 18.1% were satisfied with the VTS. Most respondents (56.7%) found that the VTS was somewhat effective, and multiple barriers were identified, such as limited time spent identifying COVID-19 cases, insufficient training of HCWs, and language differences between the HCWs and patients.

To evaluate the effectiveness of the VTS, a study of all MERS-CoV cases in a referral center was conducted from 2014 to 2017. The study showed that MERS could not be distinguished from other respiratory infections based on risk factors and clinical features [11]. Hence, the clinical scoring does not predict MERS infection. Thus, all patients with nonspecific symptoms in the MERS-endemic area will have to be isolated until MERS can be ruled out using PCR testing [11].

Some study participants believed that training HCWs was a crucial component in overcoming the reported barriers to effective VTS application. A study on 5 triage nurses and 30 emergency medical technicians showed a significant difference in the effectiveness of the triage system after training [13]. Moreover, the time spent identifying COVID-19 cases also plays a vital role since the time needed for the symptoms to appear differs from one person to another depending on his immune system; many patients remain asymptomatic [14]. Finally, communication is critical in providing high-quality healthcare services and improving patient satisfaction and health. In the case of one study, a focus group was combined with interviews to elicit the views of healthcare professionals and interpreters at one tertiary pediatric hospital in the United Kingdom. The study revealed the significant impact of language barriers and the need to consider how communication could be improved [15]. Language barriers have been associated with an increased risk of misdiagnosis, poorer understanding of and adherence to prescribed treatment, lower patient satisfaction, lower quality of care, increased risk of experiencing adverse events, poor management of chronic disease, and poorer health outcomes [15].

We included both open-ended and close-ended questions in our survey to better understand participants’ opinions toward the VTS [16-18]. In addition to participants’ suggestions for improving the system, analysis of this feedback provided insights into both the advantages and disadvantages of the VTS. The largest proportion of HCWs complained that the VTS had unclear pathways; other participants mentioned unclear checklists, inadequately trained staff, and time consumption as the main disadvantages of the VTS. However, some participants found the system effective at organizing, reducing crowds, triaging, and following up on emergency cases at a lower cost, as well as dealing with COVID-19 cases. The only suggestions included were that only specialized nurses and doctors should work in the VTS; and increase the training provided to the VTS staff.

The present study found the following factors to affect VTS satisfaction: occupation, department, and nationality. Satisfaction is a multidimensional concept. For example, people or users of a service can be satisfied with one aspect of care rather than another [16,19]. Standard dimensions incorporated in standardized satisfaction measures used in healthcare settings include interpersonal manner, technical quality, accessibility and convenience, finances, efficacy and outcomes, continuity, physical environment, and availability [20]. The provider satisfaction surveys used in this study were specifically developed based on identified stakeholder needs and the nature of the triage service. 

Study limitations included having a small sample size, being limited to two hospitals, and focusing only on providers’ satisfaction. Hence, a more extensive study is needed to assess patient and provider satisfaction.

Based on the study results, the study team recommended several modifications to enhance the effectiveness of the VTS and increase satisfaction among HCWs, patients, and policymakers. These modifications include specialized training for VTS personnel, efficient time management, and organization. To improve patient experience, it is suggested to provide linguistic assistance to patients with language difficulties and use technology to guide patients through the process. To raise awareness about VTS benefits, community engagement programs and educational campaigns are recommended.

## Conclusions

Our study findings indicated that most ED participants had a neutral outlook on their satisfaction with the VTS and a neutral perspective on the effectiveness of VTS, viewing it as only somewhat effective. Reported weaknesses and key obstacles to the successful implementation of the VTS included language barriers. and insufficient training for HCWs, and unclear VTS pathways. The reported strengths of the VTS included its effectiveness in reducing crowds and identification of COVID-19 patients.

## References

[1] Robertson-Steel I (2006). Evolution of triage systems. Emerg Med J.

[2] Dong SL, Bullard M (2008). Emergency department triage. Evid-based Emerg Med.

[3] Falzone E, Pasquier P, Hoffmann C (2017). Triage in military settings. Anaesth Crit Care Pain Med.

[4] Dippenaar E (2019). Triage systems around the world: a historical evolution. Int Paramed Pract.

[5] Martins HMG, Cuña LD, Freitas P (2009). Is Manchester (MTS) more than a triage system? A study of its association with mortality and admission to a large Portuguese hospital. Emerg Med J.

[6] Mirhaghi A, Mazlom R, Heydari A, Ebrahimi M (2017). The reliability of the Manchester Triage System (MTS): a meta-analysis. J Evid Based Med.

[7] Zachariasse JM, Seiger N, Rood PP (2017). Validity of the Manchester Triage System in emergency care: a prospective observational study. PLoS One.

[8] Beveridge R, Clark B, Janes L (2023). Implementation guidelines for the Canadian Emergency Department
Triage & Acuity Scale (CTAS). CJEM.

[9] (2023). Prehospital Canadian triage & acuity scale: prehospital CTAS paramedic guide, version 2.0. https://www.health.gov.on.ca/en/pro/programs/emergency_health/edu/docs/ctas_paramedic_guide_v2_0_en.pdf.

[10] Lababidi HMS, Alzoraigi U, Almarshed AA (2021). Simulation-based training programme and preparedness testing for COVID-19 using system integration methodology. BMJ Simul Technol Enhanc Learn.

[11] Alfaraj SH, Al-Tawfiq JA, Gautret P, Alenazi MG, Asiri AY, Memish ZA (2018). Evaluation of visual triage for screening of Middle East respiratory syndrome coronavirus patients. New Microbes New Infect.

[12] Al Harbi IS, Gupta SK (2019). Use of visual triage in the early identification and isolation of acute respiratory infection cases for the control of hospital outbreak/infection in reference to Middle East respiratory syndrome- corona virus (MERS CoV). Int J Med Public Health.

[13] Ghanbarzehi N, Balouchi A, Sabzevari S, Darban F, Khayat NH (2016). Effect of triage training on concordance of triage level between triage nurses and emergency medical technicians. J Clin Diagn Res.

[14] McCoy K, Peterson A, Tian Y, Sang Y (2020). Immunogenetic association underlying severe COVID-19. Vaccines (Basel).

[15] Williams A, Oulton K, Sell D, Wray J (2018). Healthcare professional and interpreter perspectives on working with and caring for non-English speaking families in a tertiary paediatric healthcare setting. Ethn Health.

[16] DD Royse, BA Thyer, D Padgett (2010). Program evaluation: an introduction. Padgett D.

[17] Slade SC, Keating JL (2010). Measurement of participant experience and satisfaction of exercise programs for low back pain: a structured literature review. Pain Med.

[18] Fontova-Almató A, Suñer-Soler R, Salleras-Duran L, Bertran-Noguer C, Congost-Devesa L, Ferrer-Padrosa M, Juvinyà-Canal D (2020). Evolution of job satisfaction and burnout levels of emergency department professionals during a period of economic recession. Int J Environ Res Public Health.

[19] Hudak PL, Wright JG (2000). The characteristics of patient satisfaction measures. Spine (Phila Pa 1976).

[20] Ware JE, Snyder MK, Wright WR, Davies AR (1983). Defining and measuring patient satisfaction with medical care. Eval Program Plann.

